# Dosimetric Characteristics of a Two-Dimensional Diode Array Detector Irradiated with Passively Scattered Proton Beams

**DOI:** 10.3390/cancers7030844

**Published:** 2015-07-30

**Authors:** Praimakorn Liengsawangwong, Nanayan Sahoo, Xiaoning Ding, MingFwu Lii, Michale T. Gillin, Xiaorong Ronald Zhu

**Affiliations:** Department of Radiation Physics, The University of Texas MD Anderson Cancer Center, Houston, TX 77030, USA; E-Mails: pliengsawangwong@ochsner.org (P.L.); nsahoo@mdanderson.org (N.S.); ding.xiaoning@mayo.edu (X.D.); mingjlii@mdanderson.org (M.L.); mgillin@mdanderson.org (M.T.G.)

**Keywords:** 2D diode array detector, proton, passive scattering

## Abstract

*Purpose*: To evaluate the dosimetric characteristics of a two-dimensional (2D) diode array detector irradiated with passively scattered proton beams. *Materials and Methods*: A diode array detector, MapCHECK (Model 1175, Sun Nuclear, Melbourne, FL, USA) was characterized in passive-scattered proton beams. The relative sensitivity of the diodes and absolute dose calibration were determined using a 250 MeV beam. The pristine Bragg curves (PBCs) measured by MapCHECK diodes were compared with those of an ion chamber using a range shift method. The water-equivalent thickness (WET) of the diode array detector’s intrinsic buildup also was determined. The inverse square dependence, linearity, and other proton dosimetric quantities measured by MapCHECK were also compared with those of the ion chambers. The change in the absolute dose response of the MapCHECK as a function of accumulated radiation dose was used as an indicator of radiation damage to the diodes. 2D dose distribution with and without the compensator were measured and compared with the treatment planning system (TPS) calculations. *Results*: The WET of the MapCHECK diode’s buildup was determined to be 1.7 cm. The MapCHECK-measured PBC were virtually identical to those measured by a parallel-plate ion chamber for 160, 180, and 250 MeV proton beams. The inverse square results of the MapCHECK were within ±0.4% of the ion chamber results. The linearity of MapCHECK results was within 1% of those from the ion chamber as measured in the range between 10 and 300 MU. All other dosimetric quantities were within 1.3% of the ion chamber results. The 2D dose distributions for non-clinical fields without compensator and the patient treatment fields with the compensator were consistent with the TPS results. The absolute dose response of the MapCHECK was changed by 7.4% after an accumulated dose increased by 170 Gy. *Conclusions*: The MapCHECK is a convenient and useful tool for 2D dose distribution measurements using passively scattered proton beams. Variations in MapCHECK’s dose response with increasing levels of total accumulated radiation dose should be carefully monitored.

## 1. Introduction

Interest in proton beam therapy has significantly increased in recent years because of its ability to spare healthy tissues beyond the range of the proton beam [1]. One of the methods of delivering proton beam therapy is the passive scattering approach, which uses proton beam scattering devices to expand the pencil beam laterally [2] and a range-modulation device to create a spread-out Bragg peak (SOBP) [3]. A compensator, also known as a bolus, is normally used to conform the dose to the distal end of the tumor volume in passive scattering deliveries. The compensator is created by the treatment planning system (TPS) based on the water-equivalent thicknesses (WET) between the patient’s external contour and the distal end of the tumor volume, with a margin along the proton ray lines. The difference between the largest value of WET anywhere in the field and the value of WET at the current position is the compensator thickness for this ray line [4]. Smearing, a process expanding the compensator to incorporate setup uncertainties and tumor motions and to ensure full lateral proton scatter, is normally used to ensure coverage of the full target volume [4,5].

Diode detectors have been used for measuring the depth of proton beam doses and the beam’s lateral profiles for years [6,7,8,9,10]. Most of the diode detectors’ measurements have been performed to ascertain relative dose distributions; however, Newhauser *et al.* used a diode for absolute dose measurements after calibrating it with a Faraday cup [7].

Pacillo *et al*. observed a 24% decrease in response after an accumulated dose of 300 Gy in a p-type silicon diode (designed for use in photon beams) because of radiation damage [9]. On the contrary, the p-type silicon diodes with low resistivity and higher doping levels did not change their characteristics with accumulated absorbed radiation doses, even after high doses from a proton beam had been accumulated [6]; it was suggested that this type of detector would be suitable for relative measurements in clinical settings [6,10,11].

Two-dimensional (2D) detectors would be very useful for quality assurance (QA) with proton therapy beams. In fact, use of a 2D ionization chamber array for proton therapy beam QA has been reported recently [12]. A 2D diode array detector, MapCHECK Model 1175 (Sun Nuclear, Melbourne, FL, USA), has been widely used for QA in intensity modulated radiation therapy (IMRT) [13], but its efficacy in proton beam therapy has not been reported. In this work, we studied the dosimetric properties of the MapCHECK^TM^ irradiated with passively scattered proton beams.

## 2. Materials and Methods

In the Proton Therapy Center Houston at The University of Texas MD Anderson Cancer Center, the passive scattering proton beams are generated by a synchrotron and the Hitachi PROBEAT delivery system (Hitachi Ltd, Tokyo, Japan). After the protons have been accelerated to the desired energy level, they are “spilled” from the synchrotron. The typical length of time per spill is 0.5 seconds and the nominal source-to-axial distance (SAD) is 270 cm for passive scattering beams [14]. The MapCHECK consists of 445 N-type diodes in a 22 × 22 cm^2^ array with variable spacing between diodes, ranging from 7.07 mm in the center to 14.14 mm in the outer ring. The active area of each diode is 0.8 × 0.8 mm^2^. The diode detectors are located 1.35 cm below the front surface of the MapCHECK, and the nominal intrinsic buildup to the detectors is 2.0 g/cm^2^, as stated by the manufacturer. Details of the construction of the diode array detector have been described previously [13].

The relative sensitivity of the diodes and absolute dose calibration were determined following the manufacturer’s recommendations as adapted for passive scattering proton beams. Briefly, the diode detectors of the MapCHECK were placed in the plane 11 cm below the isocenter for the relative sensitivity determination. A 250 MeV beam with a range of 25.0 cm and a SOBP of 10 cm was used to determine the relative sensitivity of the diodes. An additional 18-g/cm^2^ buildup (total buildup was ~20 g/cm^2^) was used to ensure the diodes were located near the center of the SOBP. A square aperture was used to create a field size of 25 × 25 cm^2^ in the plane of the isocenter or of 26 × 26 cm^2^ in the plane of the diode detectors. 

**Figure 1:**
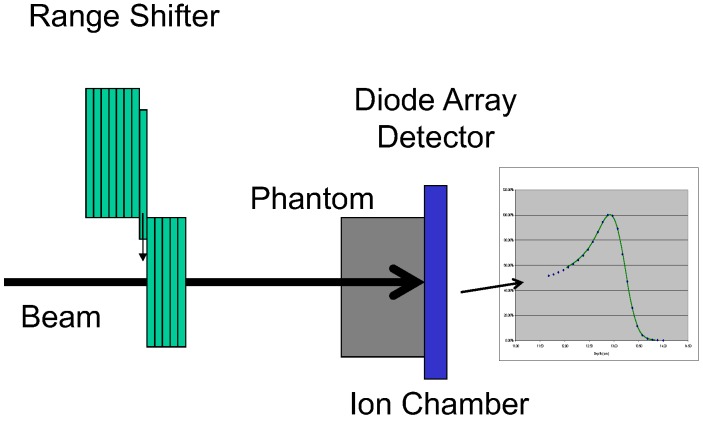
Schematic of the setup for measuring pristine Bragg curves using a range shift method.

At the end of the proton range, the LET of a proton beam increase quite rapidly [15]. To determine if there was any LET dependence of the diode detector response, we compared the PBCs measured with the MapCHECK with those from a parallel-plate ion chamber (IC) (Markus chamber, Model 23343, PTW-Freiburg, Freiburg, Germany) using a range shift method. In this method, the different thickness of the range shifter was placed in the beam after each measurement. The detectors were irradiated with the same amount of radiation in each measurement. The readings of the detectors were recorded as a function the thickness of the total range shift. The passive scattered proton beam with the field size of 10 × 10 cm^2^ without compensator was used for the measurements, as shown in Figure 1. The nominal proton energies used for these measurements were 160, 200, and 250 MeV. Each range modulation wheel (RMW) was fixed and the proton beam passed through the thinnest part of the RMW to generate the PBC. To improve the precision of MapCHECK measurements, we averaged the readings of seven diodes in the center of the MapCHECK positioned in the center of the field with uniform dose. These average readings were plotted against the thickness of the range shifter as the BPCs. The WET of the MapCHECK intrinsic buildup was determined by shifting the PBCs measured with the MapCHECK to match the ones measured by IC. The match was determined by the distal side of the PBCs for all three energies (160, 200, and 250 MeV). Special attention was paid to the distal 80% (R80) and 90% (R90). The shift required to match the MapCHECK measured PBCs to the IC measured ones was the intrinsic WET of the MapCHECK buildup.

The diode detector response as a function of source-to-detector distance (SDD) from 240 cm to 310 cm was compared with the results of the Markus chamber under the reference calibration condition, at a depth of 23.5 cm in a solid water phantom irradiated with a 250-MeV proton beam with a range of 28.5 cm and a SOBP width of 10 cm. A change of SDD from 240 to 310 cm would alter the instantaneous dose rate by approximately 40%. Therefore, the SDD dependence of the diode response was an indirect test of the instantaneous dose rate dependence.

The linearity of the MapCHECK diodes was also tested and compared with the Markus chamber under the same conditions. Other dosimetric quantities, including output factors, SOBP factors, and range shift factors, were measured and compared with those from the Markus IC. The SOBP factor is defined as the change in dose/MU with change in SOBP width relative to the reference SOBP width, which is 10 cm for the three energies (160, 200 and 250 MeV), studied in this work [16]. The range shifter factor is defined as the change in dose/MU with the thickness of the range shifter added to the beam relative to that with no range shifter [16].

The dose response of the MapCHECK was calibrated prior to each use. The MapCHECK was irradiated at the center of SOBP under the reference calibration condition as discussed above. The raw readings of the diodes were used to calculate the response, dose/count(reading), as a function of the accumulated proton dose.

Open-field dose profiles were measured with the MapCHECK and compared with the results obtained by a Pinpoint cylindrical ion chamber (Model 31014, PTW-Freiburg, Freiburg, Germany). 2D dose distributions for clinical fields with compensators were measured with the MapCHECK and compared with the TPS results.

## 3. Results and Discussion

### 3.1. Results

**Figure 2:**
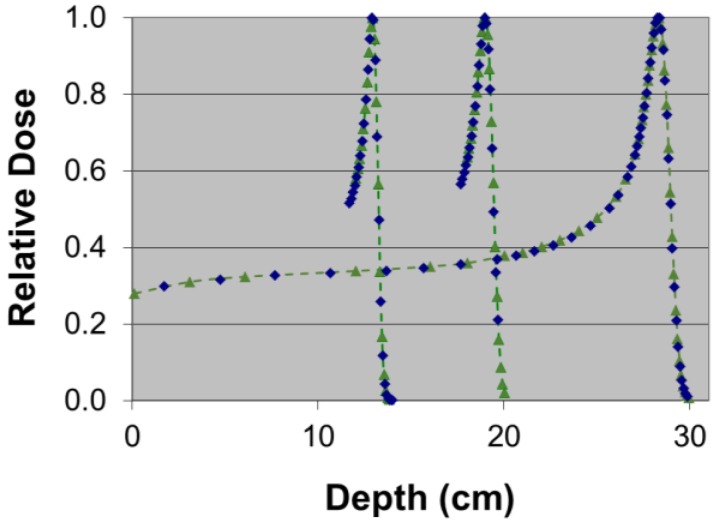
Pristine Bragg curves measured with the MapCHECK (diamonds) and ion chamber (triangles and dashed lines).

**Figure 3:**
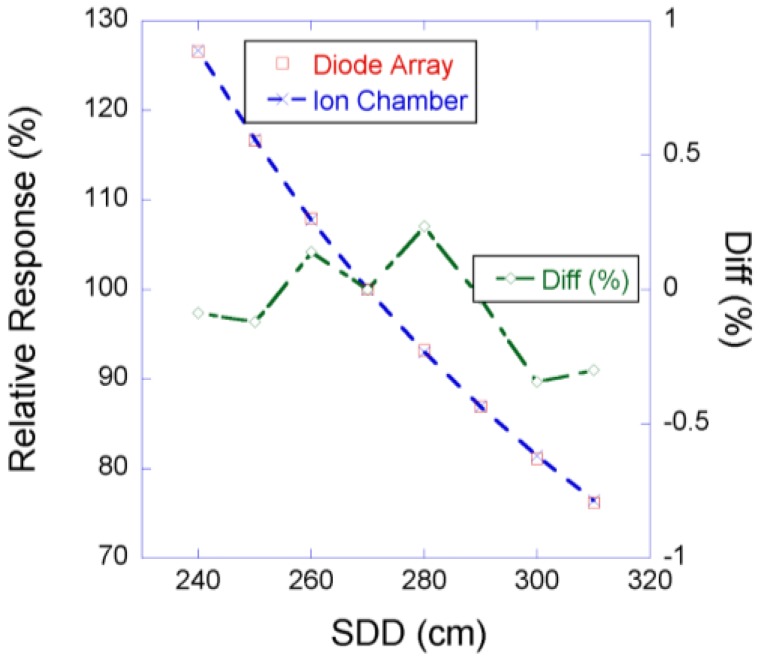
Source to detector distance dependence to test inverse-square of diode response (Diode array: open circles; ion chamber: crosses and dashed line).

**Figure 4:**
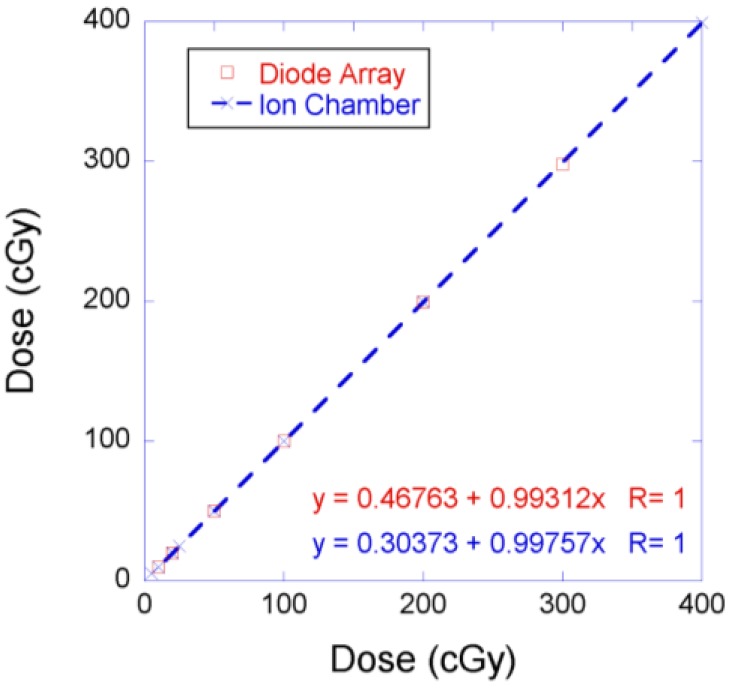
Linearity comparison between the MapCHECK and ion chamber (Diode array: open circles; ion chamber: crosses and dashed line).

**Figure 5:**
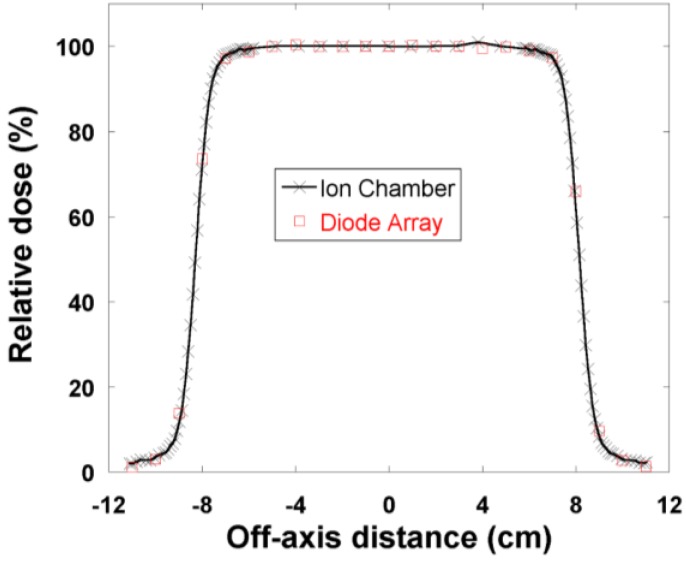
Example of a comparison between open field dose profiles measured at a depth of 23.5 cm with the ion chamber (crosses and line) and MapCHECK (open circles) for a field irradiated with a 250-MeV proton beam with a range of 28.5 cm, spread out Bragg peak of 10 cm, nominal source to surface distance of 270 cm, and aperture physical size of 14.8 × 14.8 cm^2^ located 8 cm above the phantom surface.

**Figure 6:**
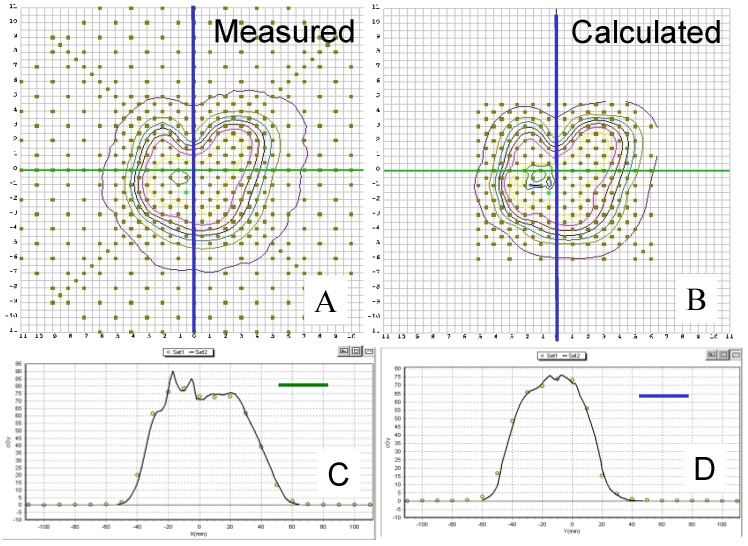
Example of comparison between dose distributions measured by MapCHECK and those calculated by the Varian Eclipse treatment planning system. 2D distribution: (**A**) Measured; (**B**) Calculated. Dose profile comparison: (**C**) along the horizontal line (green); (**D**) along the vertical line (blue); Measured: yellow points; Calculated: solid lines.

**Figure 7:**
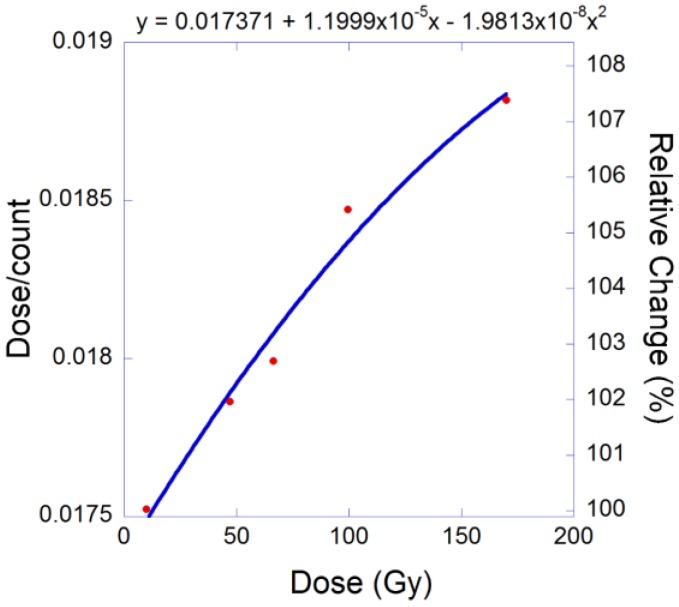
Absolute dose-per-count variation with total accumulated dose.

Figure 2 shows the PBCs measured by the MapCHECK to be virtually identical to those measured by the Markus chamber for 160-, 180-, and 250-MeV proton beams. The WET of the MapCHECK buildup was 1.7 cm determined from PBCs for three energies in Figure 2, which was less than the 2.0 cm value specified by the manufacturer. The SDD dependence of the MapCHECK was within ±0.4% that of the ion chamber, as demonstrated in Figure 3. The linearity of the MapCHECK was within 1% of the ion chamber values for MUs in the range between 10 and 300 MU, as shown in Figure 4. All other dosimetric quantities for 160-, 180-, and 250-MeV proton beams, including relative output factors, SOBP factors, and range shift factors, were within 1.3% of the ion chamber results, as listed in Table 1, Table 2 and Table 3. Figure 5 shows an example comparison of lateral dose profiles between MapCHECK and IC measurements for a 250-MeV proton beam. Figure 6 is an example of a patient field measured at a depth of 6.4 cm with the MapCHECK and calculated by the Eclipse TPS. This was a right-posterior oblique field used as one of the boost fields for the base of skull. The treatment field had a range of 14.5 cm and a SOBP width of 11 cm. The gamma analysis [17] for this example had 94% passing rate using 3%-3 mm criteria. The absolute dose response of the MapCHECK is plotted in Figure 7 with accumulated proton dose of 170 Gy. The change in the detector response by 7.4% was observed.

**Table 1:** Output factors measured by MapCHECK and ion chamber.

Energy (MeV)	Output Factor Ion Chamber	Output Factor MapCHECK	Diff (%)
250	1.000	1.00	-
180	0.886	0.881	−0.6
160	0.809	0.802	−0.9

**Table 2:** SOBP factors measured by MapCHECK and ion chamber.

Energy (MeV)	SOBP (cm)	Ion Chamber	MapCHECK	Diff (%)
250	4.0	1.256	1.241	−1.2
	16.0	0.901	0.896	−0.6
180	4.0	1.336	1.338	−0.2
	12.0	0.940	0.940	0.0
160	4.0	1.394	1.382	−0.9
	12.0	0.928	0.926	−0.3

**Table 3:** Range shift factor measured by MapCHECK and ion chamber.

Energy (MeV)	Rang Shift (cm)	Ion Chamber	MapCHECK	Diff (%)
250	4.8	0.962	0.965	0.3
180	3.0	0.920	0.919	−0.1
160	2.9	0.878	0.881	0.4

### 3.2. Discussion

The MapCHECK 2D diode array detector can reproduce, within experimental uncertainties, all dosimetic quantities measured in this work by an ion chamber for passively scattered proton beams except the variation of dose response of the detector with the accumulated total proton dose.

Near the end of the proton beam range, the LET of a proton beam undergoes significant changes. Potential LET dependence is a concern for diode detectors. Our results in Figure 2 indicate that no LET dependence was observed for diodes in the MapCHECK for the 250 MeV beam, resulting in nearly identical PBC to the measured by the ion chamber. The difference in MapCHECK buildup WET values determined by our measurements and those determined by the manufacturer (1.7 cm *vs.* 2.0 cm) suggests that one should not use the vendor-specified value without independent verification.

The response of the MapCHECK is independent of SDD in the range of 240 to 310 cm (30 cm above and 40 cm below the isocenter) as shown in Figure 3. This indicates that within the clinically relevant distances, there is no need for concern about the change in response of the diode detector due to variations in instantaneous dose rates. The results in Figure 4 show that the response of the MapCHECK is also linear within the clinical range of MUs (10 to 300 MU).

We found excellent agreement between all dosimetric quantities, including relative output factors, SOBP factors, and range shift factors, measured with the diode array detector and those measured by the ion chambers. In addition, lateral dose profiles in square fields without compensators also showed agreement with the ion chamber results. Our results also demonstrate that the diode array detector is capable of measuring 2D dose distributions for clinical fields with a compensator (Figure 6).

The clear advantage of MapCHECK is its smaller size of detector (<1 mm) compared to the 2D ion chamber array detector a diameter of 4.5 mm [12]. However, variation of the MapCHECK dose response with the accumulated total dose is a concern. Thus, performing absolute dose calibration before each measurement session is necessary; this procedure is simple and easy to perform. The change of dose response is presumably caused by radiation damage to the diode detectors used by MapCHECK. The increase in dose/count(reading), as shown in Figure 7, as a function accumulated cannot be attributed to the so called “quenching effect” in the Bragg peak with increasing dose. Each point in the Figure 7 was obtained from irradiation under the identical reference condition, that is, the detectors in the MapCHECK were placed at the isocenter plane at the depth of 23.5 cm (in the center of SOBP) in a solid water phantom irradiated with a 250-MeV proton beam with a range of 28.5 cm and a SOBP width of 10 cm. There was no increasing dose in the Bragg peaks throughout the measurements for data presented in Figure 7. The relative sensitivity of diodes, however, should be carefully monitored and a stringent periodic QA program should be developed. The authors would suggest a QA program that includes a frequent calibration of relative sensitivity of the diodes (e.g., monthly), depending on the usage of the detector. The manufacturer should consider different types of diodes with smaller variation of sensitivity with the accumulated dose, such as p-type silicon diodes with low resistivity and higher doping levels as suggested by Grusell and Medin [6] for proton applications.

The variation of the MapCHECK dose response with the accumulated total dose did not affect the results of relative measurements in Figure 2, Figure 3, Figure 4, Figure 5, Figure 6 and Figure 7 and Table 1, Table 2 and Table 3 or in routine clinical use. This was because the total accumulated dose in each session of measurement was not large enough to have significant impact on the result. For example, the measurements performed for BPCs for 250 MeV used large accumulated dose, 10 Gy. Based on Figure 7, 10 Gy would result in about 0.4% change in diode response, which was within the measurement uncertainties. 

Scanning beam proton therapy has become popular in recent years because of its ability to deliver intensity modulated proton therapy. Although there are many centers continuing use of passive scattering beams to treat their patients, many new centers have scanning beam only. One of the limitations of the current study is that it only focused on passive scattering beam. We believe that MapCHECK can also be useful for measuring 2-D dose distribution of the scanning proton pencil beams. However, this remains to be validated and will be pursued as a separate project.

## 4. Conclusions

We have demonstrated that the MapCHECK 2D diode array detector is dosimetrically nearly equivalent to the ion chamber for passively scattered proton beams. This device should serve as a valuable tool for measuring dose distributions of passively scattered proton beams. Care should be taken to monitor the variation of dose response of the detector with the accumulated total dose. The manufacturer should explore different types of diodes as alternatives for proton beam applications in order to reduce the effect of radiation damage to the detector.
